# Proactive Coping and Mental Health Among Airline Pilots During China's Regular Prevention and Control of COVID-19: The Role of Perceived Stress and Social Support

**DOI:** 10.3389/fpubh.2022.890145

**Published:** 2022-05-17

**Authors:** Quan Xu, Yaoliang Wu, Ming Ji, Mengyun Wang, Chao Pan, Jie Ma, Xuqun You

**Affiliations:** ^1^School of Psychology, Shaanxi Normal University, Xi'an, China; ^2^Shaanxi Provincial Key Laboratory of Behavior and Cognitive Neuroscience, Xi'an, China; ^3^School of Computer Science and Technology, Xidian University, Xi'an, China; ^4^Flight Department, China Eastern Airline Ltd. Anhui Branch, Hefei, China

**Keywords:** mental health, proactive coping, perceived stress, social support, airline pilots

## Abstract

Mental health has always been a prominent public health concern, and it has become more important in the wake of the COVID-19 pandemic. The mental health of airline pilots plays a significant role in their occupational health and overall performance. It is also vital for ensuring the safe operation of aircrafts. Therefore, it is crucial to identify the factors that may improve the mental health of pilots. This study investigates the relationship between proactive coping, perceived stress, social support, and mental health among airline pilots during China's regular prevention and control of COVID-19. Using a sample consisting of 285 Chinese commercial airline pilots, we tested a moderated mediation model to explore whether, how, and when proactive coping affects the mental health of pilots. The results show that proactive coping has a direct and positive effect on pilots' mental health, as well as an indirect effect on mental health through its influence on perceived stress. Social support was found to weaken the relationship between perceived stress and mental health. It also weakened the indirect relationship between proactive coping and mental health through perceived stress. These findings advance our understanding of the underlying mechanisms that affect the mental health of pilots. It also provides empirical evidence for effective mental health interventions for airline pilots during regular prevention and control of COVID-19.

## Introduction

The 2019 coronavirus (COVID-19) pandemic is a public health crisis of global concern. At present, the pandemic has been brought under control in China. China has moved into the stage of regular prevention and control of COVID-19 (1), with the focus on guarding against imported cases and preventing a resurgence of the outbreak at home. Even so, there are still new sporadic instances of local and imported cases being seen in China, and the overall situation of the current global pandemic remains severe. The COVID-19 pandemic not only damages individuals' physical health but also harms their mental health (2). Recently, researchers have noted that the civil aviation industry has experienced substantial operational problems caused by COVID-19, and pilots' mental health may be negatively affected as a result (3, 4). Specifically, uncertainty, the pressures of managing the epidemic in flight, and financial concerns due to reduced flights during COVID-19 may be damaging the mental health of airline pilots (3). The vocational requirements of airline pilots also pose mental health risks, including the stresses of flight safety, and fatigue from flight schedules (3, 4). The mental health of airline pilots plays an important role in their occupational health and overall performance. It is also important for the safe operation of the aircraft (5). As an example, on March 24, 2015, Germanwings flight 4U 9525 crashed and killed 150 people. The investigation revealed that the co-pilot suffered from depression and deliberately manipulated the plane to crash (6). Consequently, it is critical to identify the factors that may affect pilots' mental health, especially in the context of the COVID-19 pandemic. However, few studies have examined this.

Existing studies have pointed out that disparities in mental health among individuals can be understood in the context of stress and coping (7). Most of the psychological literature on coping tends to focus on reactive coping, which is the effort that people make to cope with stressful events that have already occurred, mitigating harm or loss (8). However, individuals do not have to wait for a threat to occur before introducing coping mechanisms. Consequently, a new coping theory that has absorbed the perspectives of positive psychology has been developed, known as Proactive Coping Theory (9). Proactive coping is a future-oriented coping style, and defined as “an effort to build up general resources that facilitate the promotion of challenging goals and personal growth” (9). Individuals who engage in proactive coping do not view future risks or potentially difficult situations as threats but as challenges. In that sense, proactive coping involves goal management with stress as an opportunity for growth, rather than as risk management. Thus, it helps to promote the mental health of individuals (5, 10). A study of breast cancer patients found that proactive coping promoted the post-traumatic growth of patients and prevented the deterioration of their mental health (10). In addition, proactive coping is an important protective factor when it comes to firefighters' mental health (11). In studies of the mental health of older adults, proactive coping has been found to relieve stress from COVID-19 (12) and play a positive role in life satisfaction (8). Accordingly, in the context of regular epidemic prevention and the control of COVID-19, pilots may benefit from engaging in proactive coping as this could help them to improve or maintain their mental health. Based on this, we hypothesize that: proactive coping is positively related to mental health in pilots (Hypothesis 1).

According to the Theory of Stress and Coping (13), stress is a dynamic process, and an individual's response to a stressor depends on their perception of the stressful experience. Perceived stress reflects an individual's psychological reaction after a cognitive appraisal of a potentially threatening or challenging event (14, 15). When faced with a stressful event, an individual who perceives more stress is more likely to have negative emotions, which will adversely affect their mental health (16). A study of black women exposed to sexual violence found that perceived stress was strongly related to symptoms of depression and post-traumatic stress disorder (PTSD) (7). Perceived stress has also been demonstrated to be an important risk factor for poor mental health among young adults (16) and older adults (17). Furthermore, proactive coping was demonstrated as an important predictor of perceived stress (18, 19). In the face of stressful events, the more proactive coping skills a person has, the lower their perceived stress level will be (20). A study of the ability of freshmen to adjust to college demonstrated that proactive coping was negatively associated with students' perceived stress (21). A study by Bui et al. (20) also found that proactive coping reduced perceived stress in a sample of Vietnamese undergraduate students. Taken together, this shows that perceived stress plays a significant role in the relationship between proactive coping and mental health. Proactive coping seems to affect perceived stress directly, and this, in turn, may directly affect mental health. Thus, we hypothesize that: perceived stress mediates the relationship between proactive coping and mental health (Hypothesis 2).

Social support is defined as “the flow of emotional concern, instrumental aid, information, and/or appraisal between people” (22). In the workplace, the support of colleagues is a very important source of social support (23). In the airline industry, in particular, the successful completion of flight tasks depends on the cooperation between crew members and colleagues (24). The stress-buffering model of social support proposed that social support can buffer the impact of perceived stress on the physical and mental health of individuals (25). In line with this model, Li et al. (26) demonstrated that high levels of social support weakened the positive relationship between perceived stress and prenatal depressive symptoms in pregnant women. Canavan et al. (27) have also found that social support moderates the effect of stressful life events on mental health. Moreover, a study of the mental health of university graduates has shown that social support moderates the relationship between the stress of looking for jobs and depression (28). Thus, the following hypothesis is proposed: social support moderates the relationship between perceived stress and mental health such that the relationship will be weaker when social support is higher (Hypothesis 3).

Taken together, we propose a moderated mediation model in which social support moderates the indirect effect of proactive coping on mental health through perceived stress. Specifically, according to the Conservation of Resources Theory, social support is considered an important social resource (29). This means that pilots with better social support will have more social resources, which may compensate for their lack of proactive coping skills, thereby helping them to alleviate perceived stress and improve their mental health (26, 27). Thus, the indirect effect of proactive coping on mental health will be less obvious because of the compensatory effect of high levels of social support. By contrast, the indirect relationship between proactive coping and mental health will be stronger when the quality of social support is low. Based on this, we hypothesize that: social support moderates the indirect relationship between proactive coping and mental health through perceived stress in such a way that the relationship is stronger when social support is lower (Hypothesis 4).

**Figure 1:**
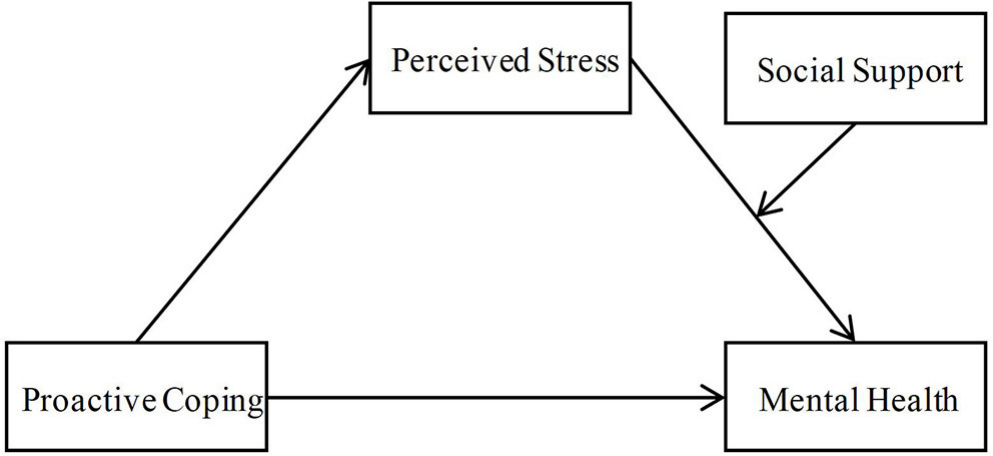
The proposed moderated mediation model in this study.

To sum up, the present study aims to explore the relationship between proactive coping, perceived stress, social support, and mental health among airline pilots during China's regular prevention and control of COVID-19. Accordingly, as illustrated in Figure 1, this study established a moderated mediation model to examine the moderating effect of social support and the mediating role of perceived stress in the link between proactive coping and pilots' mental health.

## Methods

### Participants

The study was conducted during the period of China's regular prevention and control of COVID-19, from September 27, 2021, to November 13, 2021. In total, 285 commercial airline pilots from China Eastern Airlines (CES) participated in the study. With the help of airline managers, all participants completed the anonymous, self-reported measures online. All participants were male, and their ages ranged from 22 to 61 years (M = 31.60, SD = 7.07). Their total flight hours ranged from 325 to 27,500 h (M = 6087.61, SD = 5655.93). Among the participants, there were 36 instructors, 87 captains, and 162 vice-captains. In total, 62.81% of the respondents were married, and 37.19% were unmarried.

## Measures

### Proactive Coping

Proactive coping was assessed using the Chinese validated version of the Proactive Coping Scale (PCS) (30), which consists of eight items. Sample items included the following: “When I experience a problem, I take the initiative in resolving it,” and “I always try to find a way to work around obstacles; nothing really stops me”. The participants were asked to respond on a 6-point Likert scale, ranging from 1 = *not at all true* to 6 = *completely true*. Higher scores on the scale indicated higher levels of proactive coping. The Cronbach's α of the scale was 0.85 in the current study.

### Perceived Stress

Perceived stress was assessed using a fourteen-item Perceived Stress Scale (PSS) (14). Sample items included the following: “In the last month, how often have you felt difficulties were piling up so high that you could not overcome them,” and “In the last month, how often have you felt that you were unable to control the important things in your life.” All items were measured using a 5-point Likert scale, ranging from 1 = *never* to 5 = *very often*. Higher scores on the scale indicated higher levels of perceived stress. The Cronbach's α of the scale was 0.87 in the current study.

### Social Support

Social support was assessed using a three-item scale from Chen and Kao (24), which measures individuals' perceptions of peer support. Sample items included the following: “In our company, colleagues can always count on each other for support”, and “In our company, colleagues are always willing to help each other.” The participants were asked to respond using a 7-point Likert scale, ranging from 1 = *strongly disagree* to 7 = *strongly agree*. Higher scores on the scale indicated higher levels of perceived social support. The Cronbach's α of the scale was 0.96 in the current study.

### Mental Health

Mental health was assessed using the five-item Mental Health Inventory (MHI-5) (31). The MHI-5 assesses symptoms of anxiety and depression (three items: feel nervous, downhearted and blue, and feel down) and psychological well-being (two items: feel peaceful, happy) in the last month. All items were evaluated using a 6-point Likert scale, ranging from 1 = *none of the time* to 6 = *all of the time*. Items 1, 3, and 5 are reversed. A higher score on the scale indicated better mental health. The MHI-5 is an effective instrument for assessing an individual's mental health status, including Chinese samples (2, 32). The Cronbach's α of the scale was 0.82 in the current study.

### Statistical Analysis

The data were analyzed with SPSS 22.0 and the supplemental PROCESS macro 3.3. PROCESS macro is a computational tool in which the user selects a preprogrammed model that corresponds to the model the user wants to examine. Moreover, PROCESS macro is provided with arguments about the roles that variables may serve in the model (i.e., independent, dependent, mediator, moderator, covariate), and various statistics including path coefficients, standard errors, *t*- and *p*-values, confidence intervals can be estimated (33).

The Cronbach's α coefficient was used to evaluate the internal consistency of every measure. Descriptive statistics and correlation analyses of the main variables were also performed. In addition, a regression-based path analysis was performed using PROCESS macro to test the mediating and moderating effects. This approach is considered more rigorous and has higher statistical power than stepwise regression techniques when testing for mediating or moderating effects, because all paths are measured simultaneously rather than step by step (34). Specially, the PROCESS Macro for SPSS (Model 4) was used to test the mediating role of perceived stress on the relationship between proactive coping and mental health, and Model 14 was used to test the moderating role of social support on the mediation path based on the bias-corrected percentile bootstrap method (5,000 samples) (34). The effect was deemed significant when the bootstrap confidence interval did not include zero (34). Simple slope analysis was performed to inspect the nature of the moderating effect.

## Results

### Descriptive Statistics and Correlations

The descriptive statistics and correlations among the main variables are presented in Table 1. Skewness values ranged from −0.69 to 0.20, and kurtosis values ranged from 2.34 to 3.26. The skewness and kurtosis values were within acceptable ranges (i.e., skewness <2.0 and kurtosis <7.0), indicating that all variables were normally distributed (35). The results of the correlation analyses revealed that proactive coping was negatively related to perceived stress and positively related to mental health. The results also indicated that perceived stress was negatively correlated with mental health. In addition, social support was negatively correlated with perceived stress and positively correlated with mental health. These significant correlations confirm the preliminary relationships between the variables predicted in our hypotheses.

**Table 1:** Descriptive statistics and correlations of the main variables.

**Variables**	**Mean**	**SD**	**Skewness**	**Kurtosis**	**1**	**2**	**3**	**4**
1. Proactive coping	4.62^a^	0.71	0.20	2.34	—			
2. Perceived stress	2.42^b^	0.52	−0.22	2.47	−0.56^**^	—		
3. Social support	5.69^c^	1.03	−0.69	3.26	0.65^**^	−0.48^**^	—	
4. Mental health	4.59^a^	0.79	−0.05	2.36	0.59^**^	−0.63^**^	0.58^**^	—

a *Range 1-6;*

b *Range 1–5;*

c *Range 1–7*.

** *p < 0.01*.

### Testing the Mediation Effect

Following the procedure to examine the moderated mediation model (36), we first tested the simple mediation model using the PROCESS Macro Model 4 (34) to examine the mediating role of perceived stress. The results are presented in Table 2. After controlling for age and total flight time, proactive coping was found to have a positive effect on mental health (Model 1: β = 0.58, *p* < 0.001), indicating a significant total effect. Thus, Hypothesis 1 was supported. Also, proactive coping had a significant negative effect on perceived stress (Model 2: β = −0.57, *p* < 0.001). Perceived stress had a significant negative effect on mental health (Model 3: β = −0.44, *p* < 0.001), and the direct association between proactive coping and mental health remained significant (β = 0.33, *p* < 0.001). In addition, the bias-corrected bootstrap analyses demonstrated that perceived stress had a significant partially mediating role in the relationship between proactive coping and mental health (indirect effect = 0.25, Boot *SE* = 0.06, 95% *CI* = [0.15, 0.40]). Therefore, Hypothesis 2 was supported.

**Table 2:** Testing the mediation effect of perceived stress on the relationship between proactive coping and mental health.

**Predictors**	**Model 1 (mental health)**			**Model 2 (perceived stress)**			**Model 3 (mental health)**		
	**β**	***t***	**95%CI**	**β**	***t***	**95%CI**	**β**	***t***	**95%CI**
Age	−0.07	−0.48	[−0.37, 0.23]	−0.14	−0.91	[−0.44, 0.16]	−0.13	−0.10	[−0.40, 0.13]
Total flight time	0.02	0.12	[−0.28, 0.32]	0.12	0.74	[−0.19, 0.42]	0.07	0.51	[−0.20, 0.34]
Proactive coping	0.58^a^	11.83^***^	[0.49, 0.68]	−0.57^b^	−11.42^***^	[−0.67, −0.47]	0.33^b^	6.17^***^	[0.22, 0.43]
Perceived stress							−0.44^b^	−8.46^***^	[−0.55, −0.34]
*R^2^*	0.35			0.32			0.48		
*F*	49.60^***^			44.41^***^			64.42^***^		

*All data are standardized*.

a *Total effect;*

b *Direct effect. Perceived stress is the mediator in Model 3. CI, confidence interval;*

*** *p < 0.001*.

### Testing the Moderated Mediation Effect

We then adopted the SPSS PROCESS Macro Model 14 (34) to test the moderated mediation. According to previous research (36), four conditions were tested: (a) the effect of proactive coping on mental health; (b) the effect of proactive coping on perceived stress; (c) the interaction between perceived stress and social support in predicting mental health; and (d) the different conditional indirect effects of proactive coping on mental health, via perceived stress, across low and high levels of social support. The results are presented in Table 3. After controlling for age and total flight time, proactive coping was found to have a positive effect on mental health (β = 0.21, *p* < 0.001). It also had a significant negative effect on perceived stress (β = −0.57, *p* < 0.001). Social support was found to have a positive effect on mental health (β = 0.20, *p* < 0.001). The interaction term of perceived stress and social support had a significant positive effect on mental health (β = 0.14, *p* < 0.001). These results supported conditions (a), (b), and (c), respectively.

**Table 3:** Testing the moderated mediation effects of proactive coping on mental health.

**Predictors**	**Model 1 (perceived stress)**			**Model 2 (mental health)**		
	**β**	***t***	**95%CI**	**β**	***t***	**95%CI**
Age	−0.14	−0.91	[−0.44, 0.16]	−0.12	−0.93	[−0.37, 0.13]
Total flight time	0.12	0.74	[−0.19, 0.42]	0.08	0.60	[−0.18, 0.33]
Proactive coping	−0.57	−11.42^***^	[−0.67, −0.47]	0.21	3.49^***^	[0.09, 0.32]
Perceived stress (PS)				−0.44	−8.55^***^	[−0.54, −0.34]
Social support (SS)				0.20	3.45^***^	[0.09, 0.31]
PS × SS				0.14	3.68^***^	[0.07, 0.22]
*R^2^*	0.32			0.54		
*F*	44.41^***^			54.06^***^		

*All data are standardized. Perceived stress is the mediator variable; Social support is the moderator variable; CI, confidence interval;*

*** *p < 0.001*.

**Figure 2:**
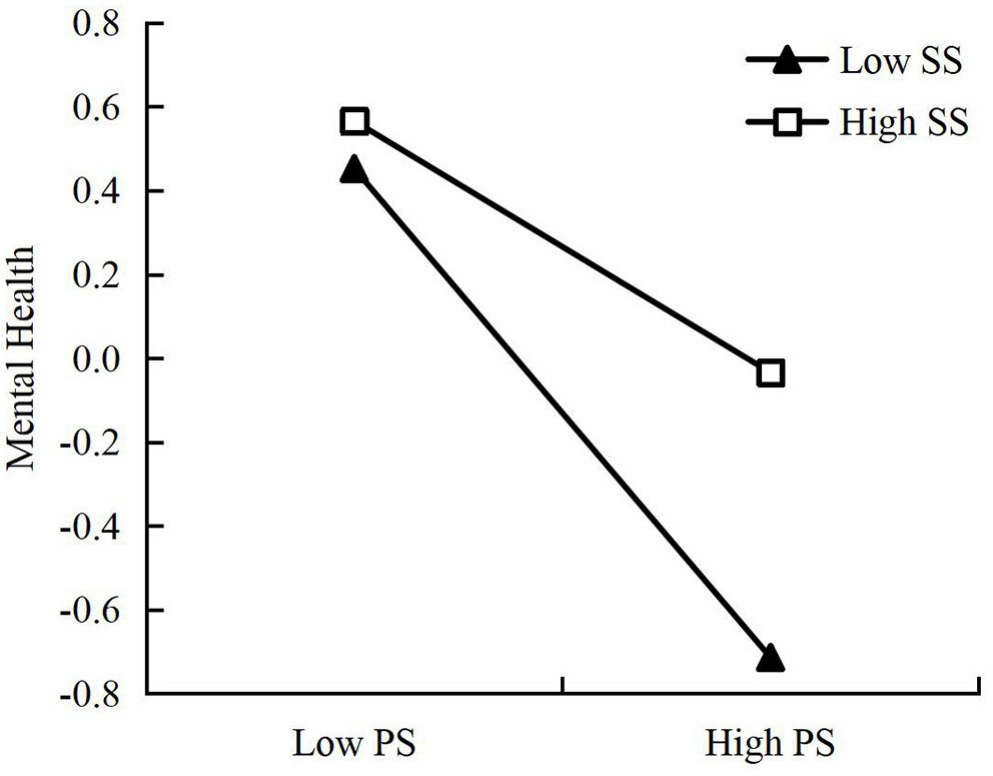
The effect of perceived stress and social support on mental health. PS, perceived stress; SS, social support. The ordinate value is standardized.

We also conducted a simple slope test to further understand the nature of the interaction effect between perceived stress and social support on mental health. As per Aiken and West (37), the scores for perceived stress and social support were divided into two groups to examine their effects: a high-scoring group (M + 1SD) and a low-scoring group (M-1SD). As shown in Figure 2, the results indicate that pilots' mental health increased as their social support increased, both in the low- and high-scoring groups for perceived stress. Moreover, the effect of perceived stress on mental health was weaker when there were high levels of social support (β = −0.30, *t* = −5.25, *p* < 0.001) than it was when there were low levels of social support (β = −0.58, *t* = −8.22, *p* < 0.001). These findings indicate that social support weakened the negative effect of perceived stress on mental health among airline pilots. Therefore, Hypothesis 3 was strongly supported.

Moreover, the bias-corrected percentile bootstrap analyses further showed that the indirect effect of proactive coping on mental health via perceived stress was moderated by social support. The results are presented in Table 4. The conditional indirect effect between proactive coping and mental health was weaker when there were high levels of social support (β = 0.17, 95% *CI* = [0.08, 0.31]) than it was when there were low levels of social support (β = 0.33, 95% *CI* = [0.21, 0.48]), thus supporting condition (d). In summary, these results demonstrate that social support moderates the relationship between proactive coping and mental health via perceived stress. Therefore, Hypothesis 4 was strongly supported.

**Table 4:** The indirect effect of proactive coping on mental health via perceived stress at different levels of social support.

**Social support**	**Effect**	**Boot SE**	**Bootstrap 95% CI**	
			**LLCI**	**ULCI**
High (M + 1SD)	0.17	0.06	0.08	0.31
Low (M − 1SD)	0.33	0.07	0.21	0.48
Difference (High-Low)	−0.16	0.06	−0.27	−0.04

*Social support is the moderator variable. SE, standard error; CI, confidence interval; LLCI, lower level confidence interval; ULCI, upper level confidence interval*.

## Discussion

The primary purpose of this study was to explore the relationship between proactive coping, perceived stress, social support, and mental health among Chinese airline pilots during China's regular prevention and control of COVID-19. The results demonstrate that proactive coping has a direct and positive effect on pilots' mental health, as well as an indirect effect on their mental health through influencing perceived stress. Moreover, social support was found to weaken the relationship between perceived stress and mental health, and the indirect relationship between proactive coping and mental health through perceived stress. These results advance our understanding of the psychological mechanisms that affect pilots' mental health. They also provide empirical evidence for effective interventions to protect the mental health of airline pilots during the regular prevention and control of COVID-19.

### Theoretical Implications

This study has demonstrated the positive direct effect of proactive coping on pilots' mental health. Pilots with higher levels of proactive coping were found to exhibit better mental health in comparison with those who reported lower levels of proactive coping. Our findings accord with previous studies of the mental health of breast cancer patients, firefighters, and older adults (8, 10, 11). Previous psychological studies of coping have tended to focus on reactive coping (8), and our study has extended the analysis of proactive coping to the field of occupational healthcare in aviation. It has focused on a future-oriented coping style, and found that proactive coping benefits the mental health of airline pilots during the regular prevention and control of COVID-19. This confirms the idea proposed by Proactive Coping Theory that individuals who engage in proactive coping do not view future risks or difficult situations as threats; rather, they view them as challenges that can facilitate personal growth and help them to improve their health (5, 10). Therefore, this study demonstrates the importance of proactive coping and highlights its potential usefulness in managing the occupational health of airline pilots during the regular prevention and control of COVID-19. Moreover, the study supplements current research into the mental health of pilots and the results of proactive coping.

This study has revealed that perceived stress appears to mediate the relationship between proactive coping and mental health. This enriches our understanding of how proactive coping influences mental health. Pilots who engage in more proactive coping can reduce perceived stress, thus improving their mental health. Therefore, our results demonstrate that proactive coping not only directly affects mental health but also indirectly affects mental health by transmitting from perceived stress. These findings are consistent with the results of prior studies of the relationship between proactive coping and perceived stress (20). They also align with studies of the effect path between perceived stress and mental health (7, 17). Although previous studies have shown that proactive coping plays an important role in improving life satisfaction, personal growth, and health (5, 8, 11), the underlying mediating effect of perceived stress had not previously been established. Therefore, the present study provides insight into the psychological mechanism and open the “black box” of the relationship between proactive coping and mental health. To our knowledge, this is the first study to prove the existence of the path of proactive coping—perceived stress—pilots' mental health. This means that proactive coping and perceived stress are significant predictors of pilots' mental health, but perceived stress is also a proximal factor that plays a more decisive role in the mental health of pilots.

This study has pointed to the notion that social support moderates the relationship between perceived stress and mental health as well as the indirect relationship between proactive coping and mental health through perceived stress. When social support is higher, the link between perceived stress and mental health, and the indirect relationship between proactive coping and mental health, are weaker compared with when there are lower levels of social support. As an important social resource (29), social support helps pilots to overcome difficulties during the regular prevention and control of COVID-19, and improves their mental health. These findings echo those related to the stress-buffering model of social support (25), and deepen our understanding of the beneficial effects of social support on the occupational health of pilots during the regular prevention and control of COVID-19. Previous research has demonstrated that social support can improve mental health directly (17), and our study has shown how social support buffers the negative implications of a lack of proactive coping on mental health as a boundary condition. Thus, our study suggests that social support can also improve occupational health in the workplace by playing a compensatory role, thereby extending the literature on social support as a boundary condition.

### Practical Implications

The present study provides several practical implications that can help managers of airlines or air transport management departments to improve the mental health of pilots, especially during the regular prevention and control of COVID-19.

First, our findings suggest that proactive coping has a significant positive effect on the mental health of pilots. Thus, one practical implication of our study is that it shows the importance of acknowledging the significance of proactive coping in managing the occupational health of pilots. Specifically, introducing a training program to develop the proactive coping abilities of pilots might be an effective way of preventing the onset of mental health problems. For example, Stauder et al. (38) have found that training employees in coping skills helps to reduce stress and improve well-being. During the regular prevention and control of COVID-19, pilots are faced with many stressors such as uncertainty, economic concerns, the stresses of flight safety and the pressures of managing the epidemic in flight (3, 4). In this case, managers may be able to promote healthy working environments, encourage pilots to adopt more proactive coping strategies, and help pilots to realize the importance of proactive coping for their mental health through training programs.

Second, the current research highlights that perceived stress partially mediates the relationship between proactive coping and the mental health of pilots. This indicates that perceived stress is a proximal factor affecting pilots' mental health. Accordingly, a potentially valuable intervention would be to place greater emphasis on pilots' perceived stress. On the one hand, training and interventions relating to the cognitive evaluation of stressors by airline pilots could reduce perceived stress (7, 14), thereby reducing its harmful impact on mental health. On the other hand, airlines could take some targeted measures to relieve pilots' stress according to the sources of stress, so as to protect their mental health. For example, airlines could design reasonable and scientific flight schedules to reduce pilot fatigue, and provide pilots with care and support in life to alleviate their financial concerns due to reduced flights (3, 4). Airlines could also carry out pilot Employee Assistance Program and psychological lectures to alleviate the negative emotions of pilots due to the pressures of managing the epidemic. In addition, airlines could stimulate pilots' inner passion for flight safety by creating positive safety climates, thereby relieving pilots' perceived safety pressure at work (39). All of these options would help to protect the mental health of pilots.

Third, this study finds that social support moderates the indirect relationship between proactive coping and mental health. This underscores the fact that social support is more effective in helping pilots with low levels of proactive coping to improve their mental health. Therefore, airline managers should recognize the importance of social support as a means of improving pilots' mental health, and they may be able to create a work environment that values social support. For instance, some researchers have investigated improving social support as an intervention to prevent and treat symptoms of depression (26, 40). We suggest that airline managers consider developing support groups for pilots to improve their mental health. This would be especially valuable for pilots with low levels of proactive coping.

### Limitations and Future Research

These findings should be considered in light of several limitations. First, this study used a cross-sectional design, which did not allow for the examination of changes in pilots' mental health at different times. This may have limited the predictive value of the research data. Future studies could use a longitudinal design to investigate the relationship between these variables and establish causality. Second, the research sample for this study consisted entirely of male airline pilots, thereby limiting the generalizability of the findings in terms of gender and occupation. Specifically, it is unclear whether the findings would be the same for different genders or whether they could be generalized to other occupations like flight attendants, air traffic controllers, and other airline employees. Therefore, future studies could use a more representative and diverse sample to examine the relationship between these variables. Third, this study found that perceived stress exerts a partial mediating effect on the relationship between proactive coping and mental health. However, there may be other psychological mechanisms that mediate the relationship between proactive coping and mental health. Thus, future researchers could examine other possible mediators.

## Conclusion

In conclusion, the present study has found that proactive coping has an important protective effect for pilots' mental health during the regular prevention and control of COVID-19. Proactive coping indirectly affects pilots' mental health by transmitting from perceived stress. The indirect relationship between proactive coping and pilots' mental health through perceived stress is weakened by social support. By answering whether, how, and when proactive coping affects the mental health of pilots, the present study has enhanced our understanding of the psychological mechanisms that affect pilots' mental health. It has also provided empirical evidence for effective interventions to help protect the mental health of airline pilots during the regular prevention and control of COVID-19.

## Data Availability Statement

The raw data supporting the conclusions of this article will be made available by the authors, without undue reservation.

## Ethics Statement

The studies involving human participants were reviewed and approved by School of Psychology, Shaanxi Normal University. The patients/participants provided their written informed consent to participate in this study.

## Author Contributions

QX and YW: conceptualization and writing—original draft preparation. QX, MW, and CP: methodology. QX, YW, JM, and XY: formal analysis and investigation. XY, MJ, and MW: writing—review and editing. QX and MJ: funding acquisition. XY and MJ: supervision. All authors read and approved the final manuscript.

## Funding

This study was supported by the National Social Science Fund of China (19BSH038) and the Fundamental Research Funds for the Central Universities of China (2020TS013).

## Conflict of Interest

JM is employed by China Eastern Airline Ltd. The remaining authors declare that the research was conducted in the absence of any commercial or financial relationships that could be construed as a potential conflict of interest.

## Publisher's Note

All claims expressed in this article are solely those of the authors and do not necessarily represent those of their affiliated organizations, or those of the publisher, the editors and the reviewers. Any product that may be evaluated in this article, or claim that may be made by its manufacturer, is not guaranteed or endorsed by the publisher.
